# Phosphorus Supplementation Recovers the Blunted Diet-Induced Thermogenesis of Overweight and Obese Adults: A Pilot Study

**DOI:** 10.3390/nu8120801

**Published:** 2016-12-09

**Authors:** Maya S. Bassil, Omar A. Obeid

**Affiliations:** 1 Department of Natural Sciences, Lebanese American University, Beirut 1102 2801, Lebanon; 2 Department of Nutrition and Food Sciences, American University of Beirut, Beirut 1107 2020, Lebanon; oo01@aub.edu.lb

**Keywords:** phosphorus, supplementation, diet induced thermogenesis, obesity, energy expenditure, energy balance

## Abstract

Diet-induced thermogenesis (DIT) is believed to be largely related to ATP production, which is dependent on phosphorus (P) availability. We aimed to test the effect of P addition on DIT of lean and overweight/obese healthy subjects. DIT was measured with or without P in 10 lean and 13 overweight/obese adults in a double-blind randomized cross-over pilot study with one week washout period. After 10 h overnight fast, resting metabolic rate, respiratory quotient, and substrate utilization were measured at fasting and every 30 min for 3 h after subjects drank a standardized glucose solution, with P (500 mg) or placebo pills. Subjective ratings of hunger and satiety were assessed before and after the end of each experiment using validated visual analogue scale (VAS) questionnaires. Overweight/obese subjects had a blunted DIT with placebo, while P supplementation induced a 23% increase in their DIT area under the curve (*p* < 0.05), which was associated with a significant increase in carbohydrate oxidation. Subjects had lower appetite following P supplementation, which was expressed as a significantly (*p* = 0.02) lower desire to eat a meal (4.0 ± 0.7 cm) compared with placebo (5.8 ± 0.9 cm). P supplementation recovers the blunted diet-induced thermogenesis in overweight and obese subjects and enhances their postprandial satiety.

## 1. Background

Diet induced thermogenesis (DIT) is the increase in energy expenditure above the basal resting rate that takes place after the ingestion of food components. It accounts for 5%–15% of total energy expenditure [1], and is the highest for protein-rich foods [2]. 

There is a large body of evidence that shows a blunted or reduced DIT in obese individuals [3,4,5,6,7]—especially those with diabetes [8]—that was normalized after weight loss [6,7]. Therefore, insulin resistance and/or insulin shortage plays a role in postprandial adenosine triphosphate (ATP) production [9], most likely by reducing the peripheral uptake of substrates needed for its production—mainly glucose and phosphorus [10]. This triggers a vicious cycle, since phosphorus depletion also appears to induce insulin resistance and glucose intolerance in animals [11]. Similarly, an inverse relationship was observed in humans between serum phosphate levels and glucose levels and homeostatic model assessment of insulin resistance index (HOMA-IR) [12,13,14]. In line with this, the addition of phosphorus to meals was shown to improve HOMA-IR in rodents [15,16]. We have also recently demonstrated that meal P supplementation improves postprandial glucose tolerance in healthy men [17]. 

On the other hand, obese subjects taking P supplementation in a weight reducing program had an increase in their basal metabolic rate [18,19]. Similar results were recently reported in rats, whereby the administration of a high phosphorus diet for four weeks induced thermogenesis by increasing uncoupling protein-1 (UCP-1) expression in brown adipose tissue [16]. However, few studies examining the effect of P supplementation of DIT in humans have been conducted. Only two studies on older postmenopausal women have revealed that the addition of P to meals increased postprandial thermogenesis in obese but not lean women [20,21]. No other study looked at the impact on younger lean or obese men and women. 

Postprandial ATP production in the liver has also been linked to signaling satiety, whereby changes in hepatic energy status are transmitted via hepatic vagal afferent activity to the central nervous system [22,23,24,25,26]. Accordingly, an analysis of the metabolic data using the Knowledge Discovery in Databases have reached the conclusion that decreased energy levels or ATP deficiency were strongly linked to the development of obesity by driving overeating and conserving energy [27]. We have also recently found that the addition of 500 mg P to different preloads led to substantial reduction in ad libitum subsequent energy intake (27%–33%) [28]. Therefore, in addition to its aforementioned role in thermogenesis and insulin sensitivity, the effect of P on food intake can explain the reported inverse relationship between P status, obesity, and metabolic syndrome [13,29,30,31,32,33]. 

More studies are warranted to investigate the relationship between P intake and DIT and satiety in the context of obesity and insulin resistance in young adults. Therefore, the objective of the present study was to test the effect of P supplementation on diet-induced thermogenesis and satiety after the ingestion of glucose solution in lean and overweight/obese healthy men and women. 

## 2. Experimental Section

### 2.1. Study Design and Participants 

The experimental design is a double blind randomized cross over design, whereby each subject was studied twice (two experimental sessions) with a minimum of 1-week washout period. A total of 10 lean and 13 overweight and obese (herein referred to only as obese) adults were recruited. Subjects were healthy, non-smokers, sedentary [34], with stable body weight for the past 3 months, not following a special diet (confirmed by multi-pass 24-h recall), and not taking any medication that affects metabolism. Subjects were asked to stop caffeine one week before the experiment.

### 2.2. Experimental Protocol

After 10 h overnight fast and after signing the consent form detailing the study’s purpose and protocol, weight, height, and body composition (Tanita BC-418, Tanita Corporation, Tokyo, Japan) were measured using standard procedures. Thereafter, resting metabolic rate (RMR) was measured using indirect calorimetry (Cosmed Fitmate, Rome, Italy). Subjects were then asked to drink a standard 75 g glucose solution (TRUTOL^®^ Glucose Tolerance Beverages, Thermo Fisher Scientific Inc., Waltham, MA, USA) with 500 mg phosphorus pills (potassium phosphate, 23% monobasic and 18% dibasic) on the first experiment and placebo pills (Cellulose) on the second one (Nutricap Labs, Farmingdale, NY, USA). Using indirect calorimetry, diet-induced thermogenesis was assessed afterwards by measuring RMR every 30 min for the following 3 h. Respiratory quotient (RQ = VCO_2_/VO_2_) was calculated at fasting and postprandially from the volumes of oxygen consumed and carbon dioxide produced. Substrate utilization—namely carbohydrate and fat oxidation—were also calculated from non-protein RQ [35].

### 2.3. Questionnaires

At the end of the experiment, subjective appetite scores were collected from the subjects, using visual analogue scale (VAS) questionnaires [36]. Moreover, habitual P intake (within the past month) was assessed for all subjects using a culturally-specific semi-quantitative food frequency questionnaire (FFQ) that included P-rich food items (both natural and processed), categorized into meat and poultry, fish and shellfish, milk and dairy products, nuts and seeds, legumes, grain products, and carbonated non-alcoholic beverages. Data from the multi-pass 24-h recall and from the FFQ were analyzed for energy and nutrient analysis using Nutritionist Pro™ Diet Analysis Software version 5.1 (Axxya Systems LLC, Woodinville, WA, USA). 

This study was approved by the Institutional Review Board of the Lebanese American University.

### 2.4. Statistics

Data are reported as Mean ± SEM. Two-tailed independent *t*-test was performed to compare data between lean and obese groups. Paired *t*-test was used for comparisons between P and placebo experiments. Repeated measures were performed to analyze within-subject results for diet-induced thermogenesis. Pearson’s correlation was used to compute the bivariate relations between BMI and P intake. Based on the effect size and SD difference from the study by Khattab et al. (2015) [17], a minimum of 10 subjects are needed to achieve 90% power with two-sided 5% significance level. All data were analyzed with the SPSS version 23 statistical package, and statistical significance was defined as *p* < 0.05. 

## 3. Results

Subject characteristics are presented in Table 1. Obese subjects had significantly higher weight, BMI, fat percentage, and resting metabolic rate (RMR) compared to lean subjects. Fat free mass (FFM) was not different between groups, and thus RMR was not corrected for FFM. Daily energy intakes estimated from multi-pass 24 h recall did not differ between groups. However, phosphorus intake estimated from the FFQ normalized per 1000 ingested calories was significantly higher in lean compared to obese subjects. Baseline characteristics of lean and obese subjects were similar between the two experimental sessions (P and placebo). 

**Figure 1:**
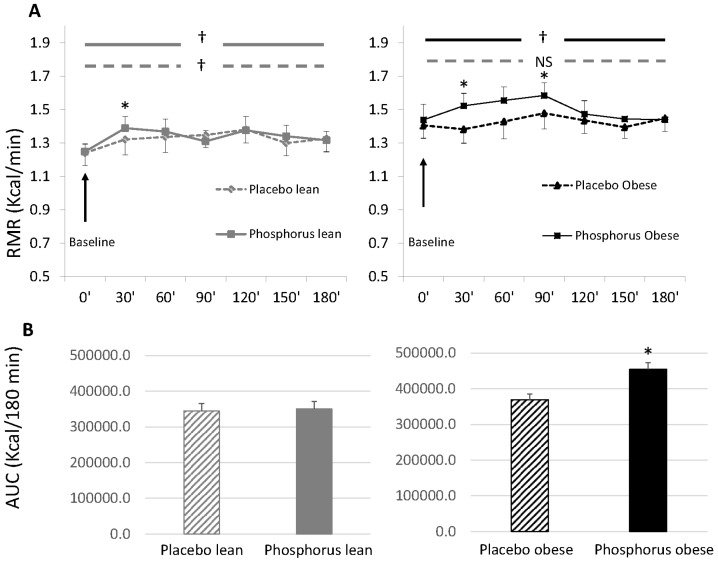
Diet-induced thermogenesis after drinking 75 g glucose solution with and without phosphorus supplementation in lean and obese subjects. (**A**) Resting metabolic rate at baseline and over 3 h (180 min) after drinking 75 g glucose solution in lean and obese subjects with phosphorus (solid lines) or placebo (dashed lines); (**B**) Total area under the curve of RMR over 3 h (180 min) after drinking 75 g glucose solution with phosphorus (solid bars) or placebo (dashed bars) in lean and obese subjects. Data are Mean ± SEM. * *p* < 0.05 vs. placebo (paired *t*-test); † *p* < 0.05: within-subjects comparison (repeated measures); NS: not significant (repeated measures); RMR: resting metabolic rate; AUC: area under the curve; SEM: standard error of the mean.

Figure 1 shows RMR measured at baseline and for 3 h after the ingestion of 75 g glucose solution in lean and obese subjects, with P or with placebo. RMR of lean subjects significantly increased above baseline with or without phosphorus (repeated measures) (Figure 1A), but area under the curve (kcal/180 min) did not differ between experiments (Figure 1B). P supplementation was associated with significantly higher RMR at 30 min in lean subjects as compared to placebo (independent *t*-test). Obese subjects had a blunted DIT with placebo, as RMR did not change from baseline over 3 h after ingestion of glucose solution (*p* > 0.05 using repeated measures) (Figure 1A), while P supplementation resulted in significant increase in RMR above baseline such that area under the curve was 23% higher compared with placebo (*p* < 0.05) (Figure 1B). Moreover, RMR of obese subjects at 30 and 90 min after glucose ingestion was significantly higher with P vs. placebo (independent *t*-test) (Figure 1A).

Substrate utilization is presented in Table 2. The obese group had significantly higher fat oxidation at baseline compared to the lean group. After drinking 75 g glucose solution, carbohydrate oxidation was significantly increased, while fat oxidation was suppressed, consistent with a significant increase in RQ. P supplementation did not affect the rate of substrate oxidation in lean subjects. On the other hand, postprandial fat oxidation in obese subjects was more suppressed in the placebo experiment compared to lean (58% vs. 36% suppression), but this was not observed with phosphorus (31% suppression). In addition, P supplementation resulted in a significant increase in carbohydrate oxidation in obese subjects.

**Figure 2:**
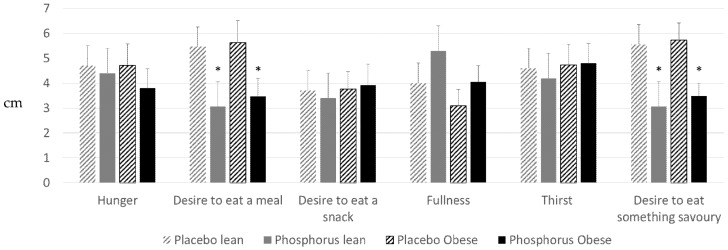
Subjective appetite scores of lean (gray) and obese (black) subjects, 3 h after drinking 75 g glucose solution with phosphorus (solid bars) or placebo (dashed bars) supplementation. Visual analog scale (VAS) questionnaires. Data are Mean ± SEM. * *p* < 0.05 vs. placebo (paired *t*-test). SEM: standard error of the mean.

Subjective appetite scores measured using VAS at the end of the experiment showed higher appetite and lower hunger with phosphorus supplementation versus placebo (Figure 2), which was statistically significant (*p* < 0.05) for the “desire to eat a meal” and the “desire to eat something savory”. 

**Figure 3:**
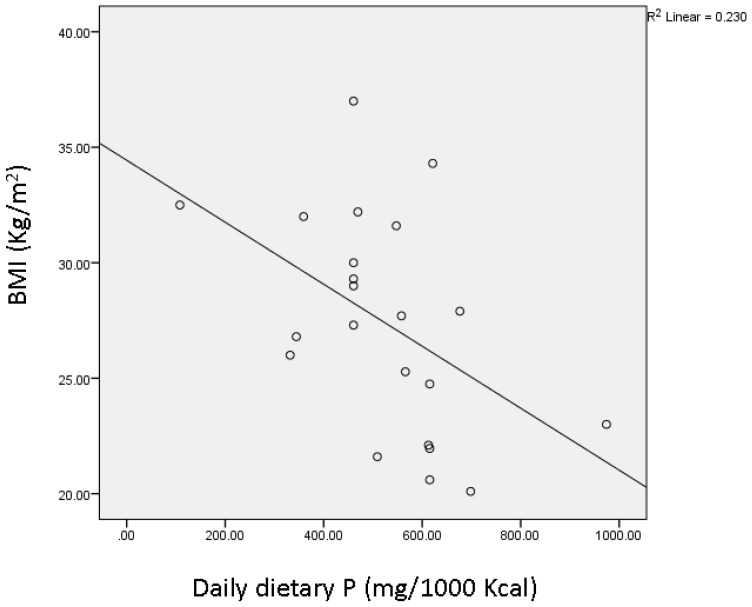
Pearson’s bivariate correlation between BMI of lean and obese subjects pooled together and daily phosphorus intake (mg/1000 kcal) estimated from food frequency questionnaire (FFQ). Pearson’s *r* = −0.48, *p* = 0.024. BMI: body mass index.

In addition, there was an inverse correlation between daily P intake (per 1000 kcal) assessed using the FFQ and BMI (*r* = −0.48, *p* = 0.024) (Figure 3) after pooling all lean and obese subjects together. 

## 4. Discussion

To our knowledge, this pilot study is the first investigating the effect of P supplementation on DIT in lean and obese young healthy men and women. Addition of P to glucose solution resulted in an increase in DIT compared with placebo in both lean and obese subjects. The increase was more pronounced in obese individuals, probably because they had a blunted DIT with placebo, whereby postprandial energy expenditure did not differ from fasting baseline. Lower DIT in obese individuals compared to lean was previously found [3,4,5,6,7], and was restored after weight loss [6,7]. This was attributed to impaired glucose tolerance and/or insulin resistance associated with excess body fat, which is improved with weight loss. On another note, chronic hypophosphatemia was shown to be inversely associated with insulin sensitivity and glucose tolerance in healthy subjects [12,13,14,37]. Conversely, a diet high in phosphorus improved HOMA-IR in db-db mice after 8 weeks [15] and healthy rats after 4 weeks [16]. We have reported similar results in humans, whereby meal phosphorus supplementation resulted in an immediate improvement in postprandial glucose tolerance in healthy subjects [17]. Since meal supply of metabolites—including that of phosphorus—is known to affect hepatic ATP production [38,39], we propose that phosphorus supplementation in the present study might have increased insulin sensitivity and subsequent phosphorus and glucose uptake by the liver, resulting in an increased ATP production and thermogenesis. Since obese subjects are more prone to insulin resistance than lean subjects, the observed effect on thermogenesis was higher in obese subjects. This is supported by our findings on substrate utilization, whereby P supplementation was associated with a significant increase in carbohydrate oxidation in obese compared to placebo, while this effect was not observed in lean. Fat oxidation in obese was also less suppressed postprandially with P compared to placebo, suggesting that P supplementation induced more carbohydrate and fat utilization, resulting in higher DIT. Nevertheless, we have not tested for insulin sensitivity or glucose tolerance in our study, and thus this needs to be further explored in future experiments. Another proposed mechanism is supported by a recent animal study showing that rats fed a high phosphorus diet (1.2%) for 8 weeks had increased thermogenesis compared to controls, which was manifested by increased UCP-1 expression in brown adipose tissue (BAT) [16]. Although thermogenesis in BAT is most accurately assessed using positron emission tomography (PET) imaging, it was reported to be directly associated with an increase in VO_2_ consumption and energy expenditure measured using indirect calorimetry in humans [40], which validates our assumptions. 

Our results are in line with those by Jaedig et al. (1994) [21] and Kaciuba-Uściłko et al. (1993) [18], which showed higher resting and postprandial thermogenesis with phosphate supplementation in older obese but not lean women. The amount of P in the supplements (~1000 mg) used in the latter studies was double that in the present study (500 mg). Moreover, we used a potassium phosphate supplement, while in the study by Jaedig et al. (1994) [21], both potassium and magnesium phosphate produced the same effect, suggesting that it is phosphate rather than potassium that is mediating the observed changes. 

Postprandial subjective appetite of lean and obese subjects was suppressed compared to placebo in the present study, 3 h after the ingestion of glucose solution with the addition of phosphorus. This is in line with our previous findings, whereby phosphorus addition to different carbohydrate preloads resulted in lower subsequent ad libitum energy intake in lean individuals [28]. Longer-term effects of P supplementation on appetite was also observed in obese individuals, 3 months after daily P supplementation (375 mg) with meals [41]. In line with this, “phosphate appetite” was previously reported among rats fed low P diet for 1 week [42,43]. We suggest that the aforementioned mechanism related to P inducing higher hepatic ATP [38,39] might also contribute to higher satiation, since increased ATP in the liver is believed to transmit afferent neural signals to the central nervous system, resulting in a suppressed appetite [22,23,24,25,26].

Interestingly, usual dietary P intake of lean subjects assessed using FFQ was significantly higher than that of obese group. This difference could not have affected baseline P, since fasting serum P is tightly controlled in healthy humans and is not affected by diet [23,44]. Therefore the effect of dietary P is only evident postprandially, when serum P rises in response to a meal, but then homeostatic mechanisms are activated to normalize it back to baseline [17]. Therefore, the observed effect of P in the present study is only due to meal P supplementation. 

Taken together, our findings support a role of P in regulating energy metabolism, and thus in the management of obesity. Indeed, an inverse correlation between P intake and BMI was observed in our study when data from lean and obese subjects were pooled together, albeit our small sample size. Consistently, this was widely reported in the literature in both animal [16] and human observational studies [12,13,29,30,31,32,33], and was supported by our recent clinical trial on obese individuals, whereby daily meal phosphorus supplementation for 3 months was associated with lower body weight, BMI, and waist circumference compared with controls [23]. This partially explains the fact that the highly available and consumed contemporary obesogenic diets are rich in fat and refined carbohydrates but low in P, while protein-rich foods that are also high in P are known to increase thermogenesis and satiety [45]. It should be noted that daily P intake in our participants (1100 ± 108 mg/day) did cover the recommended dietary allowance for P (RDA = 700 mg/day) [46], consistent with usual reported current intakes with modern diets that are rich in processed foods [47]. However, when expressed per kcal of ingested energy, reported usual P intake in the present study and in the literature (0.5–0.6 mg P/kcal) is significantly lower than that found in traditional diet regimens rich in natural unprocessed foods (1 mg P/kcal) [47]. Interestingly, these latter diets are associated with lower BMI, which supports the argument that the modern diet that is high in fat and sugar lacks sufficient P needed to process the extra ingested energy. 

Limitations of the present study include that it is a pilot study lacking assessments of potential mechanism(s) of action—especially regarding insulin sensitivity, glucose tolerance, hepatic ATP, and objective measures of appetite. Strengths include our original findings with respect to the P effect on DIT in lean and obese healthy adults and the cross-over double-blinded nature of the design that reduces bias. Future studies are warranted to replicate results in longer-term studies with large sample size and to investigate mechanism(s) of action. 

## 5. Conclusions

Phosphorus supplementation increases postprandial thermogenesis in lean and obese adults, with a more pronounced effect among obese subjects. Phosphorus also promotes fullness in obesity. Our findings set the stage for meal P supplementation or fortification as a promising obesity treatment at moderate levels (500 mg P per meal), which are far below the upper safe level of intake (4 g) [43]. 

## Figures and Tables

**Table 1:** Subject characteristics.

	Lean (*n* = 10)	Obese (*n* = 13)
Age (years)	21 ± 1	22 ± 1
Sex (M/F)	5/5	5/8
Weight (kg)	71.6 ± 6.5	89.0 ± 1.9 *
Height (cm)	173.8 ± 4.8	170.8 ± 2.2
BMI (kg/m^2^)	23.0 ± 0.9	30.6 ± 0.8 *
FFM (kg)	57.5 ± 5.3	56.5 ± 2.5
Fat mass percentage (%)	20.0 ± 2.3	36.3 ± 2.1 *
Baseline RMR (kcal/min)	1.25 ± 0.08	1.44 ± 0.05 *
Daily energy intake (kcal) ^†^	2256 ± 163	2521 ± 160
Daily dietary phosphorus (mg/1000 kcal) ^‡^	615.3 ± 56.49	460.5 ± 39.04 *
Daily dietary calcium (mg/1000 kcal) ^†^	415.4 ± 58.7	438.4 ± 31.2

Data are Mean ± SEM; * *p* < 0.05 vs. Lean (Independent *t*-test); ^†^ using multi-pass 24 h recall; ^‡^ using food frequency questionnaire. BMI: body mass index; FFM: fat free mass; RMR: resting metabolic rate.

**Table 2:** Baseline and postprandial substrate utilization.

	Lean		Obese	
	Placebo	Phosphorus	Placebo	Phosphorus
Carbohydrate oxidation				
Baseline (mg/min)	86.5 ± 12.4	77.4 ± 4.8	90.6 ± 5.9	87.6 ± 3.1
Postprandial (mg/min)	177.9 ± 13.3	182.0 ± 14.4	178.1 ± 10.5	207.6 ± 10.4 ^†^
% Change from baseline	121 ± 17	134 ± 10	107 ± 10	139 ± 12
Fat oxidation				
Baseline (mg/min)	100.3 ± 8.5	106.5 ± 6.6	125.2 ± 6.4 *	123.7 ± 3.7 *
Postprandial (mg/min)	73.5 ± 4.6	75.3 ± 5.3	79.6 ± 4.5	85.3 ± 3.9
% Change from baseline	−36 ± 7	−30 ± 1	−58 ± 9 *	−31 ± 2 ^†^

Data are Mean ± SEM; * *p* < 0.05 vs. Lean (Independent *t*-test); ^†^ *p* < 0.05 vs. placebo (paired *t*-test). RQ: respiratory quotient (VCO_2_/VO_2_).
